# Promoting recovery in daily life: study protocol for a randomized controlled trial

**DOI:** 10.1186/s40359-021-00591-w

**Published:** 2021-06-02

**Authors:** Dorota Reis, Alexander Hart, Dirk Lehr, Malte Friese

**Affiliations:** 1 Saarland University, Campus A2 4, 66123 Saarbrücken, Germany; 2 Leuphana University, Lüneburg, Germany

**Keywords:** Recovery, Occupational stress, Work-related rumination, Psychological detachment, Intervention, Measurement bursts

## Abstract

**Background:**

Work-related stress shows steadily increasing prevalence rates and has tangible consequences for individual workers, their organizations, and society as a whole. One mechanism that may help offset the negative outcomes of work-related stress on employees’ well-being is recovery. Recovery refers to the experience of unwinding from one's job when not at work. However, employees who experience high levels of work-related stress and are thus particularly in need of recovery tend to struggle to switch-off. Due to the detrimental effects of this prolonged and sustained mental representation of job stressors, interventions promoting recovery may contribute to improvements in employees' mental health.

**Methods:**

In this randomized, waitlist controlled trial, we will investigate the effectiveness of two 6-week online training programs (cognitive behavioral and mindfulness-based). The sample will include employees working at least part-time during regular work hours. Besides the pre-post-follow-up assessments, the trial will include measurement bursts with the goal of examining the underlying mechanisms. We expect that both interventions will reduce work-related perseverative thinking (PT) compared with the waitlist control groups (primary outcome). Also, we expect that both interventions will result in similar improvements, but the underlying mechanisms will differ (process outcomes). In the cognitive-behavioral intervention group, we expect that the main mechanism responsible for lower PT levels will be an increase in recovery experiences across time. In the mindfulness-based group, we expect that the main mechanism responsible for lower PT levels will be an increase in facets of mindfulness across time.

**Discussion:**

In the present study, we will investigate mechanisms underlying assumed changes in work-related PT in great detail. Besides evaluating the overall effectiveness of the two interventions in terms of pre-post-follow-up changes, we will look at the underlying processes at different levels—that is, within days, within weeks, across weeks, and between individuals. Accordingly, our study will offer a fine-grained approach to investigating potential determinants, mediators, and moderators of the processes that may, in the end, be responsible for work-related strain. From a public health perspective, if effective, the online training programs may offer valuable, low-threshold, and low-intensity interventions for a broad range of occupations.

*Trial registration* German Clinical Trials Registration: DRKS00024933. Registered prospectively 7 April 2021. https://www.drks.de/drks_web/navigate.do?navigationId=trial.HTML&TRIAL_ID=DRKS00024933

## Background

### Recovery from work-related stress

Work-related stress refers to a relationship between the employee and their work environment that is appraised as taxing or exceeding their resources and thus endangering their well-being [1]. Work intensity index measuring exposure to work demands shows a stable and high prevalence of demands such as quantitative demands, work pace, and emotional demands over the last few years [2]. The European Commission estimated that work-related stress affects 55.6 million employees in the European Union [3]. Twenty-five percent of them feel stressed for all or most of the time they spend at work [2]. In 2018, a calculation of the overall costs of work-related stress at a national and pan-European level revealed a (substantial) range from US$221.13 million to $187 billion [4]. These numbers are not without consequences. Work-related stress is associated with a number of consequences for employees' physical and psychological health. Such consequences include increased risk of coronary heart disease [5], metabolic syndrome [6], and mental health issues such as depression [7].

Given these numbers and healthcare-related costs, research has aimed to identify protective individual and organizational factors that may buffer the impact of work-related stress. One of the individual protective factors is an employee’s ability to recover from work. Recovery refers to the experience of unwinding from one's job during the time that is not spent at work [8]. The concept captures various mechanisms that counterbalance employees' reactions to job demands and help them maintain work-related well-being and health [9]. The psychophysiological systems are activated during work in response to job demands. Such reactions to job demands are functional in the first place. They are adaptive for the completion of work chores. But during the time that is spent not working, these reactions should return to a baseline level, that is, a level that appears in a situation in which no particular work demands are made on the individual [10]. Hence, whereas short-lived reactions are adaptive, prolonged and recurring experiences might be potentially harmful. Recovery has been identified as a mediating process between an individual’s responses to stressful work-related experiences and mid- and long-term mental health. Here, recovery is the mechanism that prevents allostatic load [11]. In this vein, recovery has demonstrated advantageous effects for reducing the negative consequences of work-related stress [for a meta-analytical review, see, e.g., 12]—and, conversely, it has shown positive relations with measures of well-being [e.g., 13–15].

Importantly, research has differentiated two complementary conceptualizations of recovery: recovery as a process and recovery as an outcome [8, 15, 16]. Studies investigating recovery as a *process* have mostly focused on the question of whether different experiences and strategies are more or less successful at improving well-being. The earliest approaches to recovery focused on examining the roles of various activities employees engage in when they are not at work. In such approaches, non-work activities are grouped into (a) "low-duty activities" that may promote recovery (e.g., socializing, physical activity) and (b) "high-duty activities" that may further impair recovery (e.g., child care) [15, 17]. Later studies have tended to focus on the experiences that underlie non-work activities, such as detachment, relaxation, mastery, and control. The roles that these experiences play in determining employee well-being have been investigated in general, and during specific time frames, such as breaks during the day [20], evenings [18], weekends [19] and holidays [21]. Moreover, several meta-analyses and reviews have shown that recovery experiences are positively related to, for example, mental health and well-being and are negatively related to measures of fatigue, exhaustion, or negative affect [12, 15, 22].

Among these recovery process variables, psychological detachment has been identified as a central recovery experience [9]. Etzion et al. [23] defined detachment as “the individual’s sense of being away from the work situation” (p. 579). Hence, detachment goes beyond purely "being physically away" from work. It implies a context-specific experience of not thinking about work-related issues and not being involved in work-related tasks [9]. Whereas this definition of psychological detachment refers primarily to the absence of something, Sonnentag and Fritz [9] emphasized that it includes "the experience of being mentally involved in any other content area" (p. S74) or even thinking of nothing in particular as is a common goal in meditative approaches. Although detachment is often examined along with the other three recovery experiences (i.e., relaxation, mastery, and control), it reflects the most central aspect in models such as the Stressor-Detachment-Model [9] and the DRAMMA-Model [where DRAMMA stands for detachment-recovery, autonomy, mastery, meaning, and affiliation; 24]. Despite their substantial bivariate correlations, the experiences represent distinguishable aspects of the recovery process and should not be used interchangeably [15]. In this study, we will focus mainly on the role of psychological detachment but will examine the other three experiences as potential process variables that may contribute to detachment.

Besides recovery processes, a different approach of looking at recovery refers to the *outcome* or the result of switching-off during non-work time. The outcome of recovery can be conceptualized in terms of psychological, physiological, or behavioral measures. For example, psychological aspects of recovery have been assessed in self-reports with constructs such as the "state of feeling recovered" [25]. Here, feelings of replenishment (e.g., feeling "recovered mentally" or "well-rested") serve as indicators of (successful) recovery processes [25] or are examined as a direct result of engaging in recovery activities [26]. Interestingly, such an outcome of recovery processes conceptualized as a "state of being recovered" demonstrated only small to moderate associations with the preceding process of detaching from work in a recent meta-analysis [15]. Moreover, detachment and the state of feeling recovered have shown differential meta-analytical effects on various measures of well-being and performance (e.g., positive affect and job performance). Based on these findings, we will include both detachment and the state of being recovered as recovery indicators in our study.

### Work-related perseverative thinking

Perseverative cognition or thinking (PT)—also referred to as repetitive negative thinking—is an umbrella term for the cognitive processes that underlie various emotional disorders. It has been defined as "the repeated or chronic activation of the cognitive representation of one or more psychological stressors" [27] (p. 114). Hence, PT is characterized by uncontrolled (i.e., at least somewhat intrusive), repetitive, and difficult-to-disengage-from thinking about concerns, problems, and experiences [28]. PT encompasses rumination (i.e., thoughts that focus primarily on the past) and worry (i.e., thoughts that are related primarily to the future). Accordingly, PT occurs before or after stressful events [27], and it extends the adverse effects of these events to times when the stressor is no longer or not yet present. These prolonged experiences of stressors are relevant for individual well-being and strain (reactions) because the sustained cognitive representations may produce physiological responses that are similar to those that occur during the stressful events themselves [29].

Work events represent one type of stressful event that is potentially relevant for PT. In recovery research, studies have investigated repetitive thinking concerning work-related issues in terms of work-related rumination [30]. Similar to the findings for "general" PT in the domain of occupational health, research has found that work-related rumination mediates (the appraisal of) stressful events at work and depressive symptoms [31] and sleep complaints [32]. Hence, in the context of recovery research, PT focusing on work-related issues should be considered a risk factor.

In this vein, work-related PT may reflect an experience that is strongly associated with—or is a form of—unsuccessful recovery (and in particular detachment). Someone who is engaging in PT about work cannot at the same time be detached from work, and someone who is detached from work cannot at the same time engage in PT about work. Despite the high degree of conceptual overlap between work-related PT and psychological detachment, correlations between the two constructs have been moderate [30, 33]. Also, at least cross-sectionally, work-related PC (measured as affective rumination) and detachment are empirically distinguishable [30].

Several scenarios may explain the empirical uniqueness of work-related PT and detachment. First, individuals may find that they are not detached but are still not experiencing repetitive and intrusive thoughts about work. This situation occurs, for example, when individuals work in the evenings or reflect positively on work-related events during their non-work time. Second, individuals may repetitively think about future events or situations at work. This type of PT refers to worrying (often combined with feelings of anxiety) but not rumination about past or more or less current events. However, future-oriented worrying is not well-represented in questionnaires that are designed to measure (affective, past-oriented) rumination. Stated differently, individuals may indeed have trouble winding down and may worry about future events, but this association will remain undetected when PT is measured in terms of rumination (that focuses on past events). Third, some instruments that assess work-related rumination by including adjectives that are indicative of activated negative affect such as nervous or angry [34] presume that employees experience activated negative states [35]. In our view, negative activation and PT should not be merged into single items. Negative activation and PT are co-occurring albeit potentially distinct experiences. Even for constructs that may co-occur in general, merging them into single items leads to measurement issues when used as a state assessment. In a given moment, individuals may experience either or only one of the relevant phenomena, thus raising questions about the response's validity, that is, whether the responses pertain to the activated negative affect, to PT, or to both.

To sum up, both psychological detachment and work-related PT capture non-work experiences that are linked to mental health and psychological well-being [15]. Considering how essential switching-off after work is and how detrimental the effects of PT are for the vast majority of the working population, it seems relevant to look for ways to facilitate detachment and reduce PT in employees. Hence, there is a need to better understand the underlying processes that impair recovery and serve to maintain work-related PT. In this way, we will be able to design new intervention approaches and refine the existing ones.

### Interventions promoting recovery and reducing work-related perseverative thinking

In 2011, Cropley and Zijlstra raised the concern that "there is a distinct lack of studies that have put forward interventions to help people to switch-off, unwind and recover post work" [36] (p. 495). Similarly, Ebert and colleagues (2015) stated that given that interventions for individuals with work-related mental health problems are more effective when work-related aspects are considered, "the limited amount of intervention research on combined interventions that target recovery by improving effective psychological detachment from work and sleep is surprising" [37] (p. 1241).

Meanwhile, various approaches have been designed and evaluated. Several interventions have successfully addressed detachment or repetitive work-related thinking directly [37, 38]. Others have focused on other constructs, such as improving boundary management [39], stress management skills [40], or mindfulness [41]. Hence, in this second group of interventions, increasing detachment or reducing PT was not the primary goal, but these interventions fostered detachment nevertheless.

A recent meta-analysis of interventions that were aimed at fostering detachment [42] stated that, on average, the existing heterogeneous intervention approaches yielded a positive small to moderate effect (*d* = 0.36) on detachment. Interventions that included multiple strategies, such as engagement in recovery activities, boundary management, mindfulness, and more general emotion regulation techniques, were the most successful. In addition, interventions lasting more than 2 weeks and with a higher dosage (in total more than 4 h) were more successful than briefer and lower dosage trainings.

### Combining web-based interventions with experience sampling methodology

In intervention research, there has been an increase in various web-based delivery formats in recent years. Web-based formats include, for example, online interventions with or without additional app support or interventions with varying levels of guidance. Such web-based approaches are generally associated with improvements in participants’ health. Phillips et al. [43] systematically reviewed 50 randomized controlled trials involving web-based formats and found treatment effects with Hedges’ *g* values ranging from 0.30 (for depression) to 0.70 (for insomnia). In recovery research, formats other than face-to-face formats (including web-based formats) yielded an average effect size of *d* = 0.45 [42].

Web-based interventions have numerous advantages. They are highly compatible with participants' daily life obligations due to the self-determined timing, duration, and location of interventions. Also, they are self-administered and are thereby mostly anonymous. These characteristics reduce the burden for individuals who have tight and varying schedules, are fearful of social evaluations, or might avoid or might not profit equally from face-to-face interventions due to social fears and specific therapy alliance problems [44].

From an evaluation perspective, web-based designs make it easier to match the frequency of the intervention with the frequency of the experience of interest [45]. In contrast to face-to-face interventions, participants in web-based interventions have access to the contents of the intervention in their daily lives and can coordinate the activities with their own daily routines. This allows participants to engage with the contents of the intervention whenever they need it most. For example, when addressing recovery, participants might look for supportive contents in the evenings after work. In web-based interventions, this is made feasible by monitoring the progress of the intervention with active and passive experience sampling measures, that is, daily or weekly self-report assessments of the fluctuating constructs combined with sensor-based data. The advantages of web-based interventions may be further enhanced in designs in which several more intensive phases of data collection (i.e., measurement bursts; [46]) are combined into one longitudinal study.

### Investigating processes

Complex intervention designs allow researchers to examine the underlying change mechanisms in more detail [47]. Given that unwinding is an experience that fluctuates strongly within individuals across days, we need intervention designs that capture these fluctuations and match the intervention's evaluation more closely with the behavior of interest [45]. With a pre-post-follow-up design, both the fluctuations and the trajectory of change remain unknown. By contrast, more frequent assessments paralleling the course of the intervention may reveal when the changes occur. For example, a recent mindfulness intervention study [48] found that changes in detachment followed a log-linear trajectory. This finding is in line with the dose–effect model in psychotherapy [49]. In essence, this model predicts the greatest improvements in the first phase of an intervention, followed by a phase of more stable (but improved) levels of detachment. Designs with additional assessments shed light on such trajectories of change and advance our understanding of both nonlinear improvements and phases that can be useful for stabilizing and reinforcing the change that is achieved.

With designs that include various measurements along the way, researchers can simultaneously analyze within-person processes and individual differences in these processes. For example, at the within-person level, negative activation (i.e., unpleasant mood and unpleasant discrete emotions, e.g., anger, sadness, or anxiety) and high tense arousal may represent the key variables that impair recovery [50] and serve to maintain PT. Negative activation (NA) may impede recovery because individuals experience and relive job stressors even after long periods of not working and might not even recover fully during sleep due to its job-related fragmentation [51]. Also, NA and both recovery and PT may be reciprocally related (i.e., not only does NA impede recovery/maintain PT, but low recovery/high levels of PT may, in turn, maintain NA). Shipp and Cole [52] argued that "considering the role of feedback loops, cycles, and spirals is a natural next step for within-person explanatory research" (p. 12). The reciprocal relations between recovery and PT on the one side and NA on the other side may create a loss cycle [53]. This sustained mental and affective arousal may, in turn, be associated with symptoms of strain, such as impaired sleep [54].

Intervention studies including intensive longitudinal assessments and measurement bursts create entirely new avenues for investigating within-person processes. Designs with multiple well-timed assessments or measurement burst designs are basic preconditions for looking at processes and mechanisms as they unfold in daily life. But it is the combination of such designs with interventions that puts the assumed processes to the test. Similar to an implementation of two experimental groups in a pre-post-follow-up design, a direct comparison of two different treatments allows for the strengthening of conclusions about the underlying mechanism and the boundary conditions that restrict or boost training effectiveness. Hence, in this trial, we will build upon previous research on recovery interventions [42] by implementing two intervention groups: one with a cognitive-behavioral focus and one with a mindfulness-based approach (for more details about the contents of the intervention, see below). So far, a similar direct comparison was planned in one study [55] but without the specific focus on within-person processes and trajectories of change.

In sum, by taking dynamic within-person processes into account, with our study, we aim to shed more light on the development, trajectory, and maintenance of recovery in daily life. Further, our complex design will allow us to examine and compare the effects of the two interventions on these processes. On the basis of previous literature [42], in both intervention groups, we expect improvements in recovery processes, negative activation, and strain measures at the daily level. Here, changes refer to both the respective levels of the constructs and their respective associations. The mechanisms responsible for these improvements may differ across the experimental groups. In the CBT-based group, we expect the mechanism responsible for reducing work-related PT to be an increase in recovery experiences because the contents of the intervention pertain to these strategies (e.g., boundary tactics and recreational activities). In the mindfulness-based group, we expect the main mechanism responsible for lower levels of work-related PT to be increases across time in affect regulation strategies (e.g., reappraisal) and the facets of mindfulness such as non-reactivity and acting with awareness.

## Research goals

The first goal of this randomized controlled trial is to compare the efficacy of two online training programs promoting recovery from work. We hypothesize that participants in the two experimental conditions (CBT-based and mindfulness-based training) will experience lower levels of work-related PT (primary outcome) after the intervention. No prediction can be made from previous literature as to which of the two interventions would be more effective. Therefore, we assume that the two experimental conditions will not differ with regard to their effectiveness in reducing the primary outcome.

The second goal of the present study is to explore the mechanisms related to recovery from work and its improvement on a daily level. Hence, using the data from the experience sampling phases of the study, we will investigate within- and between-day variables and processes that may account for the overall effect of the intervention—and, in addition, may further qualify the expected effect.

## Methods/design

### Study design

In this protocol, we describe a four-armed randomized controlled trial with measurement bursts (i.e., periods of intensive longitudinal data collection) conducted to compare the efficacy of two 6-week online interventions for recovery from work. We will randomly assign the participants to one of four conditions: (a) experimental condition 1: CBT-based techniques, (b) experimental condition 2: mindfulness-based techniques, (c) waitlist condition 1: CBT-based techniques, (d) waitlist condition 2: mindfulness-based techniques. To monitor progress for the assessments, we will maintain a parallel structure for all four groups, meaning that the waitlist control groups will respond to the same questionnaires in all phases of the study. We will invite all participants via email to complete the pretest (Phase 1) and the first measurement burst (comprising four daily assessments from Monday through Friday plus one assessment on Saturday morning). This first week of intensive assessments represents Phase 2. After Phase 2, participants in the experimental groups will immediately receive access to the online interventions. During the 6 weeks of the interventions in both experimental groups, participants in all groups will be prompted once a week to provide 1 day of experience-sampling measures (four daily assessments, Phase 3). Approximately 7 weeks after Phase 2, participants will begin the second week of intensive longitudinal assessments (similar to Phase 2) followed by the posttest (Phase 4). Following Phase 4, individuals in the waitlist control groups will receive access to the online training programs in either the CBT-based or mindfulness-based form—depending on their allocation at the start of the study. Finally, Phase 5 will take place 3 months after the end of Phase 4 and will again include 1 week of intensive longitudinal assessments and a follow-up assessment (similar to Phase 4).

### Participants and recruitment

The study will be conducted with employed volunteers recruited through newspapers, flyers, and social media. To estimate the power to detect an assumed meta-analytical pre-post effect of *d* = 0.36 [42], if such an effect exists in the population, we ran a Monte Carlo simulation. Our analyses for the primary outcome involve a Latent Change Model (see below for more details). The simulations showed that for a two-tailed test with 90% power and a priori alpha level of 5%, we would need *N* = 136 participants in each experimental group to be able to detect a pre-post difference of approximately *d* = 0.35 (in our case represented by the intercept of the latent change factor) if it exists in the population.

In addition, for the analyses of the dynamic associations with the measurement bursts, previous studies have shown that the power to detect a relatively small standardized indirect effect of 0.1 was = 0.81 for ICC ≥ 0.10 with *J* ≥ 100 clusters with a within-cluster sample size of *n *_*j*_ = 20 [56]. Because our research questions refer in part to the upper levels of the hierarchical data, we will need a sample size that will allow us to estimate unbiased parameters for the mediation models at the person-level of analysis as well. Li and Beretvas [57] showed that a multilevel SEM required at least 80 clusters to circumvent convergence issues.

Considering all the analyses' requirements and assuming a drop-out rate of up to 20% after Phase 3 and up to an additional 20% after Phase 4, we aim to recruit 600 participants (i.e., 480 in Phase 4 and 384 in Phase 5). The analyses will be conducted using all available data (either with Full Information Maximum Likelihood or Bayesian estimation). To be eligible for this study, participants must (1) be 18 years or older, (2) be working regular working hours (no shift work) at least part-time, (3) not be in psychotherapeutic or psychiatric treatment, and (4) be smartphone users with mobile internet access. In addition, participants must be able to read and write in German.

**Fig. 1:**
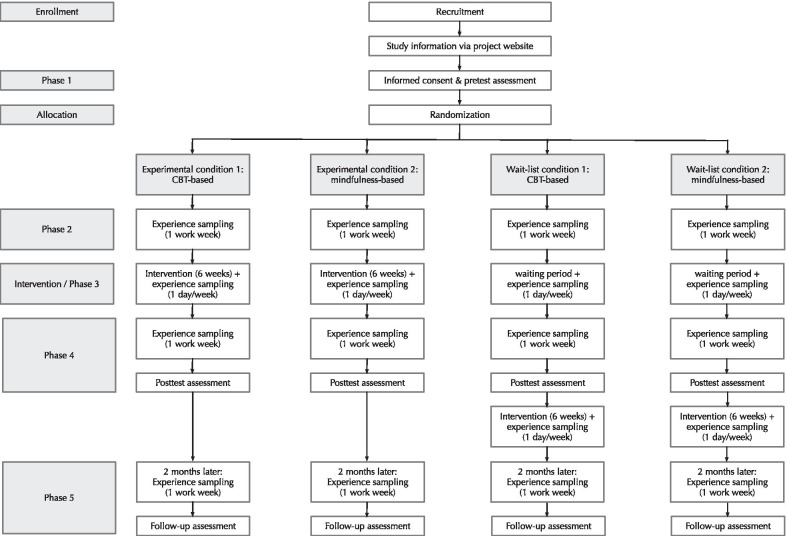
Study design

Individuals interested in participating will be able to register on the project's website, freiraum-im-kopf.de. The website contains information about the study's aims, procedure, assessments, data protection policy, and informed consent. The informed consent form explains that individual data will remain confidential. That is, all records that contain names or other personal identifiers, will be stored separately from study records identified by code number. We inform that data will be published in an anonymized form in a repository parallel to the publication. Also, participants will be assured that they may withdraw from the study at any time. A researcher not involved in the study will randomly allocate participants to one of four study conditions (see Fig. 1) using computer-generated random numbers. We plan to oversample the experimental conditions to include two thirds of all participants using block randomization. This procedure ensures an equal random distribution of the individuals into all four groups. We will ensure allocation concealment, as the randomization code will not be available to the trial investigators until the participants have completed all baseline measurements.

Considering the high participant burden, to keep participants involved in the study, we will use a bonus system to reward participation. Participants will receive €10 for completing the posttest and another €10 for the follow-up. In Phases 4 and 5, they will receive 50 cents for completing all measures at each (intensive longitudinal) measurement occasion. In addition, to motivate participants to complete as many measures as possible, participants can enter a lottery. The prizes include 10 vouchers worth €20, 20 vouchers worth €40, and 20 vouchers worth €50.

### Procedure

The overall procedure is shown in Fig. 1. The study will combine a longitudinal design with three experience-sampling phases (measurement bursts). After completing the pretest (Phase 1), participants will begin the first 5 days of the experience sampling (Phase 2). In this week, we will assess all work-related and personal variables in daily life (for an overview of all variables, see section on assessments below). During the experience-sampling phases, participants will be prompted once each morning, once in the (late) afternoon, once during the evening, and once directly before going to sleep. The morning and afternoon sessions will take approximately 2 min, whereas the evening and night sessions will take about 5 min each.

Following Phase 2, participants in the intervention groups will begin with either the CBT-based training or the mindfulness-based training. Because both interventions will last for 6 weeks, we will implement the contents of the interventions in both groups sequentially. Both training programs can be considered low-dose interventions [42]. Based on the literature, we do not expect training-related harms or adverse events. However, the study will monitor such adverse effects, and the participants may contact the research team via email at any time during the study.

### Interventions

#### CBT-based training

Overall, the elements of the CBT-based training program are built on the mechanisms suggested in the DRAMMA-model [24]. The model emphasizes the roles that detachment, relaxation, autonomy, mastery, meaning, and affiliation play in determining well-being. Training will begin with psychoeducative kick-off lessons. Here, we will focus on goal attainment, explain rituals for the transition from work to non-work time, and make suggestions about how to find spare time for relaxing activities. Participants will continue by reflecting on their situation in terms of stressors and values (e.g., meaning or affiliation). We will prompt participants to rediscover and reevaluate their values. Furthermore, we will include behavior-change techniques that directly address PT and sleep problems.

#### Mindfulness-based training

The mindfulness-based training program is built on the concepts and techniques of mindfulness-based interventions [58]. Ideas taken from approaches such as mindfulness-based stress reduction [59] may help employees set cognitive-emotional boundaries and disengage from work. Mindfulness practice may raise awareness of work-related thoughts and improve individuals' abilities to come back to the present (non-work) moment. Accordingly, training will begin with psychoeducative elements on shifting attention toward—and remaining anchored in—the present moment. It will further elaborate on the differences between “doing” and “being” modes of mind and formal and informal mindfulness practices. Formal mindfulness practice is fostered by following along with guided exercises and meditations. Alongside the formal meditations, the training program will offer recommendations for implementing informal practices in daily life and actively breaking maladaptive routines. Participants will learn to increase their self-compassion and to explore negative and positive feelings with curiosity. They will then practice noticing but not ruminating about work-related issues. Instead, they will explore how to distance themselves from feelings and thoughts, such as worries about future-related work issues.

### Intervention content, key features, and core themes

The CBT-based and mindfulness-based interventions have different contents. However, with the same goal of facilitating recovery, the two approaches have several parallel key components. The parallel features are related to the delivery of the intervention (e.g., audio recordings and quizzes) and the type of content that has demonstrated high efficacy in recovery interventions. Specifically, both of our interventions include techniques for boundary management, engagement in recovery activities, sleep improvement, and emotion regulation strategies.

#### Psychoeducation

Both interventions include psychoeducative elements. We will invite participants to explore the determinants of stress and well-being from a cognitive-behavioral or mindfulness-focused perspective. Consequently, at least one third of each week’s module will be dedicated to psychoeducation, increasing participants' knowledge on the topic.

#### Audio-guided exercises

The majority of the modules will involve listening to audio instructions ranging from meditations to podcasts, where the speakers discuss specific psychoeducational topics or give examples from everyday life. The scripts for the audio content are designed to form a bond between intervention and participant by addressing the listener directly on several occasions. Relying on audio in addition to written instructions will further allow participants to focus on topics dynamically because they will be able to change modalities according to their preferences, available time, or environment on a particular day.

#### Active and reflective elements

In both interventions, we will ask participants to engage in certain activities, such as setting their relaxation goals for the next week or completing a short quiz. Thereby, participants will be able to reflect on their progress and revisit their answers in an earlier training period as they proceed through the program. Revisiting their own responses and ratings may enhance commitment and promote self-monitoring and self-reflection.

#### Implementing micro-interventions

Beyond the CBT-based and mindfulness-based strategies provided in the interventions, the training sessions will focus on fostering thoughtful and mindful practices in daily life. In each module, participants will be given suggestions for implementing short interventions in their everyday lives. These micro-interventions will include, for example, eating mindfully or generating implementation intentions for pursuing daily recovery goals.

#### Improving adherence

If participants do not log in to the training website for 10 days, they will automatically receive a reminder email asking them whether they are experiencing any problems with the program. Responses will be handled by the research team individually. In addition to the 1 day of experience sampling that will take place each week, in the questionnaires we will also include suggestions for exercises provided in the respective week of the training program.

### Assessments

Assessments in the study will include (1) outcome assessments, (2) process assessments, (3) smartphone sensing, and (4) behavioral and self-reported compliance on the training website. Table 1 provides an overview of all assessments. In the following section, we describe the different types of assessments.

**Table 1:** Measures

	Pretest	ESM	Post-Test	Follow-Up
Intake				
Demographics	X			
Main Outcome Assessment				
Work-related Perseverative Thinking	X	X	X	X
Secondary and Process Outcome Assessment				
Recovery Experience Questionnaire [62]	X	X	X	X
State of Being Recovered [63]	X	X	X	X
Positive Work Reflection [64]	X	X	X	X
Three‐Dimensional Work Fatigue Inventory [65]	X	X	X	X
Standardized Sleep Inventory [66]	X	X	X	X
Life Engagement [adapted from 67]	X	X	X	X
Multidimensional Mood Questionnaire [68]	X	X	X	X
Multidimensional State Mindfulness [69]		X		
Five Facet Mindfulness Questionnaire [70]	X		X	X
Negative Activation [based on 71]	X	X	X	X
Perceived Stress Scale (German version) [72]	X	X	X	X
Affect Regulation [based on 73]	X	X	X	X
Further Outcome and Control Variables				
Big Five Inventory [74]	X			
Trait Self-Control [75]	X			
Utrecht Work Engagement Scale [76]	X	X		
Appreciation at Work [77]	X	X		
Emotional Resources [78]	X	X		
Autonomy [79]	X	X		
Emotional Demands [80]	X	X		
Home Demands (self-made)	X	X		
Home Resources (self-made)	X	X		
Self-Control Demands [81]	X	X		
Unfinished Tasks [32]	X	X		
Interoception [82]	X	X		
Data Quality (self-made)		X	X	X
Situational Questions		X		

#### Outcome assessments

We will evaluate the effectiveness of both interventions by comparing the respective levels of the outcome variables at pretest, posttest, and a 3-month follow-up. The pretest will take place before the first week of experience sampling and the intervention. The posttest will take place in the week after the intervention. The follow-up will take place 3 months after the posttest to evaluate whether changes in work-related PT remained stable over this more extended period or reverted to their pre-intervention levels. We will ask the participants to evaluate all primary and secondary outcomes using a time frame of 2 weeks.

The primary outcome in these pretest, posttest, and follow-up assessments is *work-related PT*. We will measure work-related PT using a combination of three worry items [60], four rumination items [28], and two brooding/rehearsal items [61]. Items have been adapted to the work context. The response scale ranges from 1 (not true at all) to 5 (very true). For a complete list of items, see the supplementary online materials on the OSF: https://osf.io/jzt6g/. This group of items has not been used in this combination as an indicator of PT before. We therefore evaluated the measure in terms of model fit and convergent and discriminant validity in a sample of 202 employees working at least part-time. A unidimensional model fit the data well (CFI = 0.98; TLI = 0.97; RMSEA = 0.07; SRMR = 0.03), and the pattern of correlations with (a) measures of similar constructs, such as affective rumination [35], detachment [62], and (b) relevant correlates (e.g., emotional demands, fatigue, stress, and affect) was meaningful and as expected. The internal consistency of the scale was good: McDonald’s ω = 92.

Secondary outcomes will be the following work and mental health variables, which are conceptually and typically empirically associated with work-related PT.

*Detachment*. We will measure psychological detachment with a four-item subscale from the Recovery Experience Questionnaire [62]. The rating scale ranges from 1 (not true at all) to 7 (very true).

*State of being recovered.* State of being recovered will be measured with four items proposed by Sonnentag and Kruel [63]. On the pretest, posttest, and follow-up questionnaires, we will ask the participants how rested and refreshed they felt during the last 2 weeks using a 5-point scale ranging from 1 (not true at all) to 5 (very true).

*Fatigue*. We will assess fatigue using nine items from the German version of the Three-Dimensional Work Fatigue Inventory [65]. This instrument differentiates between physical, mental, and emotional work fatigue with parallel items assessing tiredness and reduced functional capacity. We will ask participants to rate their fatigue using a 6-point scale ranging from 1 (never) to 6 (always).

*Sleep quality.* Sleep quality will be assessed with the sleep quality subscale from the Standardized Sleep Inventory [66]. Participants will be asked to rate their sleep with three adjectives (good, undisturbed, ample) ranging from 1 (never) to 7 (always).

*Affective well-being*. We will operationalize affective well-being in terms of good mood and (low) tense arousal with four items taken from the Multidimensional Mood Questionnaire [68]. Participants will rate their mood using adjectives such as pleasant, content, tense, or nervous on a 7-point scale ranging from 1 (not at all) to 7 (very much so).

*Negative activation.* Negative activation will be measured with five adjectives that are based on the 12-point circumplex core-affect model [71]. Adjectives include affective attributes such as irritated, upset, or agitated. We will apply the same 7-point scale as already used for the measurement of affective well-being ranging from 1 (not at all) to 7 (very much so).

*Stress.* Stress perceptions will be assessed with a 4-item German version of the Perceived Stress Scale [72]. The stress items will be answered on a 5-point Likert scale ranging from 0 (never) to 5 (very often). We will use the overall score as an indicator of the individual stress level.

Also, the pretest, posttest, and follow-up assessments will include several other variables, such as personal and work characteristics. Table 1 presents all constructs, and further details are provided in the Codebook on the OSF at https://osf.io/jzt6g/.

#### Process assessment

The measurement burst design will include various phases of intensive longitudinal assessments. In Phases 2, 4, and 5, participants will receive links to self-reports four times per day from Monday morning through Saturday morning. We will prompt participants in the morning, directly after work, at 8 pm, and before bedtime. The assessments will include items on work-related variables (e.g., job demands and resources, work engagement), mental health variables (e.g., fatigue, sleep quality, stress, recovery), and personal characteristics (e.g., affect regulation, negative activation, personal resources, and working hours). All items refer to the state aspects of the constructs. Table 1 presents the measures, and the project's Codebook on the OSF repository offers more details.

During the 6 weeks of the intervention, we will implement 1 day of experience sampling each week with a total of five assessments (beginning on Tuesday at bedtime followed by four assessments on Wednesday). The measurements will parallel those in Phases 2, 4, and 5.

#### Smartphone sensing

We will acquire sensor data from participants' mobile phones. While participants fill out the daily questionnaires, we will obtain data for the acceleration and orientation of the device. These passive sensor data may provide incremental information about individuals' internal states such as arousal or fatigue [83] and serve as a proxy for data quality. Hence, the use of additional sensor-based assessments will establish a multimethod assessment approach.

#### Behavioral and self-reported engagement

A key discrepancy between face-to-face interventions and digital self-help eHealth/mHealth training programs is the absence of scheduled appointments. This lack of social interaction requires participants to plan their own intervention schedule, complete the exercises conscientiously, and identify which content areas need to be reiterated. Failure to do so (e.g., due to low commitment) might ultimately lead to attrition [84].

Although researchers tend to agree that engagement plays a significant role in the effectiveness of self-help interventions, the determinants of compliance in eHealth/mHealth and its effects on effectiveness are largely unexplored [85]. Nevertheless, differences in engagement may partly explain the heterogeneity in effect sizes found for interventions that are comparable to each other in terms of content, delivery format, duration, dosage, and target population. Therefore, we will repeatedly assess participants’ behavioral adherence throughout the training period on the dimensions of frequency, intensity, time, and type [85].

### Data analyses

Our research goals refer to different levels of the data. Besides evaluations of the interventions’ efficacy, one part of our research involves hypotheses on the within-day level. By contrast, others focus on the same processes between days and several weeks. We will address the complexity of our data by applying various statistical approaches.

To evaluate changes across time in primary and secondary outcomes, we will use the pretest, posttest, and follow-up measures. These measures represent indicators of latent variables estimated separately in the two experimental groups and the two waitlist control groups combined. Using the latent variables (latent states), we will set up Multigroup Latent Change Models (with baseline-change and neighbor-change variants). Latent Change Scores from (a) pretest to posttest and from pretest to follow-up (baseline-change) and (b) from pretest to posttest and from posttest to follow-up (neighbor-change) will provide information about the effect sizes from pre to post and on the maintenance of the effects from posttest to follow-up [86]. To estimate the models, we will use the Bayes estimator as implemented in Mplus [87]. We will determine the convergence diagnostic [88], evaluate potential convergence issues by doubling the number of iterations, and visually inspect histograms for all parameters and the trace plots for the Markov chains. For the difference between the pre-post change scores in both experimental groups, we will set up an informative prior with a normal distribution, a mean of zero, and a small variance, that is, *N*(0, 0.010). This prior corresponds to the assumption that the change scores in the two groups should be equal, implying similar efficacy between the two interventions. A small variance around the mean of the prior will reflect our confidence that the difference between the change scores will be minimal but will allow for small differences [89]. This distribution implies that 95% of the standardized differences in the means ranges from -0.2 and 0.2. Accordingly, the efficacy of the two interventions will be considered equal if the standardized difference in the latent change scores based on the posterior distribution lies within a range of -0.2 and 0.2.

We will use the assessments paralleling the interventions to estimate a Growth Model separately in each group. To account for potential nonlinear growth over time, we will implement free time scores when estimating the slope growth factor in each group. The time score in the first week will be fixed to zero (centering point), whereas the time score in the sixth week will be fixed to one. Hence, the mean of the slope growth factor will represent the average rate of change from Week 1 to Week 6.

The Dynamic Structural Equation Modeling (DSEM) [90] approach will be applied to the measurement burst part of the study. DSEM is suitable for addressing the particular issues of (a) intensive longitudinal data, (b) autoregressive and cross-lagged within-person effects, and (c) different time-lags (between and within persons). DSEM combines four modeling techniques: multilevel modeling, time-series modeling, structural equation modeling, and time-varying effects modeling. The multilevel part of DSEM will allow us to model the correlations that are due to individual-specific effects. The time-series part of DSEM will enable us to model the correlations that are due to the proximity of observations. The structural equation modeling part will be based on correlations between different variables. The time-varying effects modeling will be based on correlations that are due to the same stage of development. This flexibility makes DSEM suitable for testing the hypotheses in our study. The postulated mediation and moderation effects will apply to the within-day level (Level 1) of analysis. To test the time course of the processes, job stressors will be measured directly after work. Negative activation, detachment, and work-related PT will be measured in the evenings. Strain variables and the state of being recovered will be assessed in the mornings. This procedure will allow us to test for autoregressive and cross-lagged associations between the constructs. Recovery strategies, mindfulness, and emotion regulation are also Level 1 variables and can be accounted for in the DSEM. Hypotheses focusing on the interventions’ effectiveness at a daily level can be implemented as a cross-level predictor. Moreover, given that the study will manipulate work-related PT, changes in the intercepts, variances, and covariances of the variables are expected and will be modeled as random effects.

In addition, the DSEM framework can account for a more realistic implementation of the time variable within a continuous-time modeling approach. This aspect of the model can account for the assumptions that the same processes will occur at the microlevel (i.e., within days) and the macrolevel (i.e., between weeks). Given that in intensive longitudinal data, uneven individual observations are most likely to occur, the analyses need to be able to account for the different time lags. Using the DSEM approach, we can examine the associations of interest by using parameters that are not biased by a violation of the assumption of equal time intervals.

For all analyses, we will use the statistical modeling programs Mplus [87] and R [91].

## Discussion

In this randomized controlled trial, we will test the efficacy of two interventions in parallel. In the interventions, we will aim to promote a mental switching-off from work. Both interventions will include components that were previously successful in facilitating recovery (e.g., boundary management, psychoeducation). But this study will extend previous research because it will consist of a combination of intensive-longitudinal and longitudinal data (measurement burst design). Our design will enable us to look at dynamic processes underlying recovery at the daily (i.e., the micro-) level nested within the macrolevel of longitudinal (i.e., across the weeks and months of the study) processes. Hence, we will examine short-term recovery dynamics that operate within longer term dynamics [9]. At the same time, we will strive to change/manipulate these dynamics to better understand the development, trajectory, and maintenance of recovery over time. Therefore, with this approach, we will be able to address crucial and as yet unanswered questions and put them to a rigorous test.

At a theoretical level, the present study will contribute to previous research on the Stressor-Detachment Model [9] and will aim to obtain more fine-grained insights into the job-stressors-recovery-strain relation and its underlying mechanisms. Negative activation and its reciprocal associations with work-related PT and detachment may represent key variables that are responsible for the recovery paradox [50] (i.e., actually impaired recovery processes when job stressors are particularly high). In addition, we will examine mechanisms by using repeated experience-sampling assessments over the course of days and weeks and will thus separate the predictors, mediators/moderators, and outcomes in time. Our study will provide insight into the trajectories of recovery in its various operationalizations as work-related PT, detachment, and the state of being recovered. Although related, they are distinct elements of recovery and should not be used interchangeably [15].

The present work realities include work digitalization and intensification and—as a consequence of the COVID-19 pandemic—a substantial proportion of employees working from home. These trends go along with (among other things) the blurring of work-life boundaries [92]. Switching-off becomes more difficult to achieve. Here, considering the relevance of recovery for mental health, low-threshold interventions may have a powerful impact on people's well-being/lives. Such interventions have the best chance of impacting employees' daily lives and natural environments. Then they can match the frequency of the intervention with the frequency of the target behavior by providing real-time support [93].

In terms of public health promotion, recovery improvements may play a crucial role in the mental health of employees. The expected increases in recovery in terms of an effect size (*d* = 0.36) are relatively small—at least when compared with effect sizes typically found in clinical studies. But interventions fostering recovery target a risk factor common in the working population, and large-scale improvements in recovery may reduce organizational and economic costs. Considering the easy implementation and applicability of online training programs for recovery in various settings, they seem like a viable option for reaching numerous employees.

## Data Availability

The datasets used and/or analysed during the current study and the statistical code will be openly available upon publication on the OSF: https://osf.io/jzt6g/.

## Abbreviations

CBT: Cognitive-behavioral therapy
DSEM: Dynamic Structural Equation Modeling
NA: Negative activation
PT: Perseverative thinking
